# FIRE AND ICE: The quest for the perfect modality in atrial fibrillation ablation

**DOI:** 10.21542/gcsp.2016.23

**Published:** 2016-09-30

**Authors:** Mohamed Sayed, Mohamed ElMaghawry

**Affiliations:** Department of Cardiology, Aswan Heart Centre, Aswan, Egypt

## Abstract

Atrial fibrillation (AF) is the most common arrhythmia in clinical practice. Catheter ablation of atrial fibrillation plays an important role in the management of AF. Radiofrequency ablation is widely used in practice all over the world. Cryoablation has emerged as an alternative method for AF ablation. The FIRE and ICE trial was a non inferiority, multicentre, randomized trial that compared between the two modalities and proved cryoablation to be non inferior to radiofrequency in terms of efficacy and safety. However, the rate of AF recurrence was markedly high in both arms of the study.

## Background

Atrial fibrillation (AF) is the most common arrhythmia worldwide and represents a significant burden on global healthcare^1^. Since its introduction in the late 1990’s, catheter ablation has gained an increasingly important role in the management of AF. The fundamental concept of AF ablation is based on recognizing that electrical foci arising from the myocardial sleeves of pulmonary veins trigger and maintain AF^2^. Therefore, pulmonary vein isolation (PVI) by different ablation techniques may help in maintaining sinus rhythm. Over the years, numerous mapping, imaging, and ablation technologies have been developed to improve the outcomes of radiofrequency (RF) AF ablation. Recently, cryoablation had been introduced as an alternative method to RF with increasing popularity among electrophysiologists^3^. Many studies have compared between the RF and cryo AF ablation modalities, however, these studies either were nonrandomized or included small number of patients^4,5^. The FIRE AND ICE trial, which has been recently published in the New England Journal of Medicine, represents the largest-to-date prospective randomized multicentre trial comparing the efficacy and safety of RF and cryo ablation in patients with paroxysmal AF^6^.

## FIRE AND ICE: Design and results

The FIRE and ICE study was a multicenter, randomized trial to compare the efficacy and safety of pulmonary vein isolation either by cryoablation or radiofrequency ablation. The study was designed to test whether cryoballoon ablation was non-inferior to RF ablation, assuming event-free 1-year survival rates of 70% with 10% non-inferiority margin, corresponding to a hazard ratio of 1.43. Inclusion criteria were symptomatic paroxysmal AF, prior antiarrhythmic drugs failure, and age from 18 to 75 years. Exclusion criteria were previous left atrial ablation or surgery, left atrium diameter larger than 55 cm, myocardial infarction or percutaneous coronary intervention with 3 months of enrollment, stroke or transient ischemic attack within 6 months of enrollment, and left ventricular ejection fracture less than 35%. The primary efficacy end point in a time-to-event analysis was the first documented clinical failure. The authors defined clinical failure as recurrence of atrial fibrillation, occurrence of atrial flutter or atrial tachycardia, use of antiarrhythmic drugs, or repeat ablation, following a 90-day period after the index ablation. The primary safety end point was a composite of death, cerebrovascular events, or serious treatment-related adverse events (e.g. phrenic nerve injury, atrioesophageal fistula, etc).

A total of 762 patients underwent randomization (378 assigned to cryoballoon ablation and 384 assigned to radiofrequency ablation). The mean duration of follow up was 1.5 years. The primary efficacy end point occurred in 138 patients in the cryoballoon group and in 143 in the radiofrequency group (1-year Kaplan Meier event rate estimates, 34.6% and 35.9%, respectively; hazard ratio, 0.96; 95% confidence interval [CI], 0.76 to 1.22; P < 0.001 for noninferiority). Subanalysis of each of the components defining clinical failure showed similar results in both arms. However, the mean total procedure time was shorter in the cryo group than in the RF group (124 vs. 141 minutes, P < 0.001), as was the left atrial dwell time (the length of time the catheter was present in the left atrium during the procedure), (92 vs. 109 minutes, P < 0.001). The mean total fluoroscopy time was shorter in the radiofrequency group than in the cryoballoon group (17 vs. 22 minutes, P < 0.001).

Regarding safety, the primary safety end point occurred in 40 patients in the cryoballoon group and in 51 patients in the radiofrequency group (1-year Kaplan–Meier event rate estimates, 10.2% and 12.8%, respectively; hazard ratio, 0.78; 95% CI, 0.52 to 1.18; P = 0.24). However, there were no tracheosophageal fistulae in the RF group and only 10 phrenic nerve injuries in the cryo group, with only one persisting as a clinical problem after 2 years follow up^6^.

**Figure 1.**
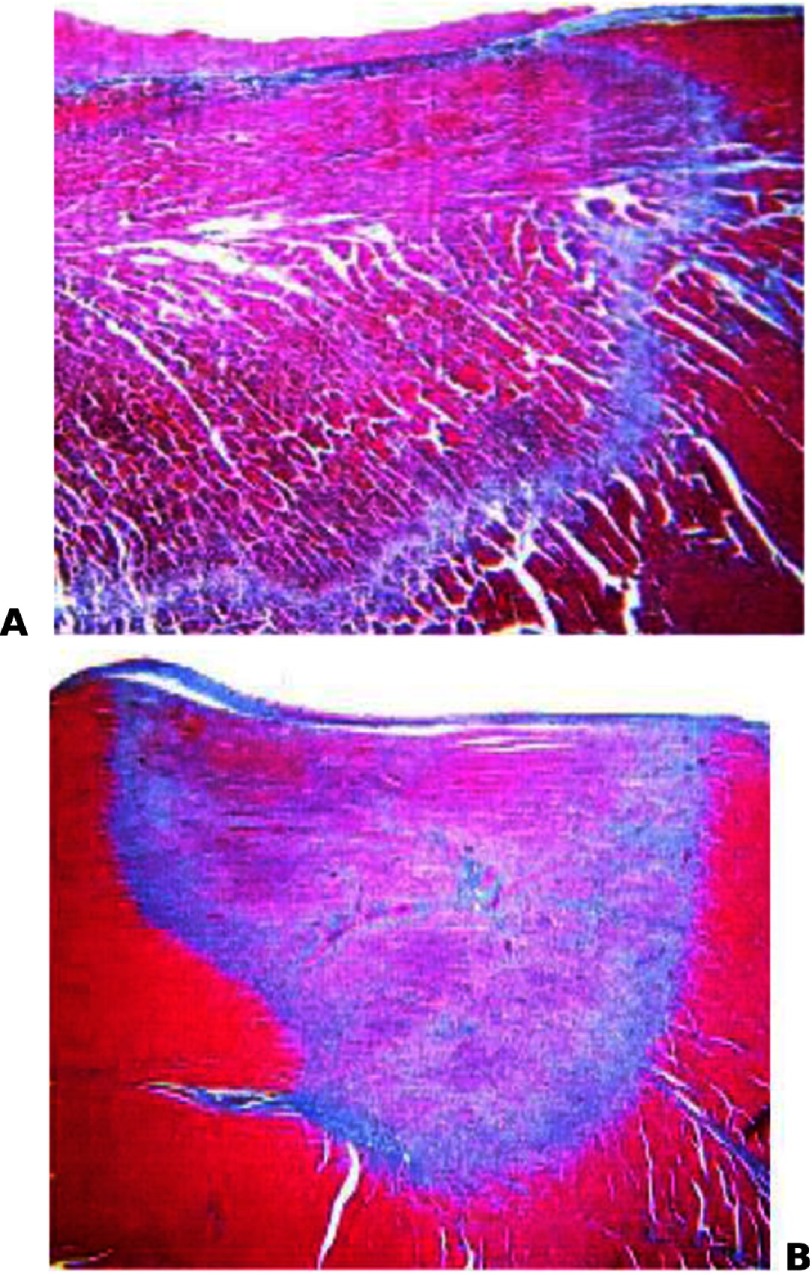
Histology of radiofrequency lesions (a), and cryo-energy (b), stained with Masson’s trichome. Source: Khairy et al., Lower Incidence of Thrombus Formation With Cryoenergy Versus Radiofrequency Catheter Ablation. Circulation, April 2003.

## Discussion and critique

The FIRE AND ICE study assessed the safety and efficacy of cryoablation versus radiofrquency ablation in patients with paroxysmal symptomatic AF^6^. PVI using radiofrequency ablation necessitates creating continuous lesion line around the ostia of the pulmonary veins which will electrically isolate the veins from left atrium^7^. RF uses high frequency alternating current (in the range of 300 kHz to 1 MHz) to create a thermal lesion. Permanent tissue necrosis occurs by direct resistive heating and indirect, passive conductive heating, when tissue temperature reaches around 55°C (Figure 1A). Many factors influence the lesion formation, including conductive cooling by surrounding fluid (blood) flow, size of ablation catheter tip, weight, angle and time of application^8^. On the other hand, permanent tissue damage occurs during cryoablation by freezing the tissue to lower than −50°C^9–12^ (Figure 1B). Potential advantages of cryoablation include the possibility of “test” freeze area of interest before reaching the permanent freezing level, thus decrease possibility of collateral damage and increase safety of the procedure. In addition, cryoablation balloon catheters will seal the pulmonary veins ostia as the stuck to tissue during freezing. This increases stability of the ablation and improves efficacy^13^.

The FIRE and ICE represents one of the largest prospective randomized controlled trials not only in comparing RF versus cryo technologies, but regarding results of AF ablation in general. The investigators recruited patients with paroxysmal symptomatic drug-refractory AF^8^. This subset of patients represent a class I indication for AF ablation according to the most recent European Society of Cardiology Guidelines^14^. Although the patient selection is in accordance with the guidelines, it may not reflect the current trend in AF ablation. Most of high volume expert centres are currently performing more ablations to patients with persistent AF, which is a Class IIB indication according to European guidelines^15–17^. The role of cryo ablations in persistent AF still needs to be investigated. Some RF advocats have previously claimed that cryo will proof to be inferior in persistent AF due to the need for ablations strategies other than PVI, such as additional lines, complex atrial fractionated electrograms (CAFÉ), ganglionic plexi, and rotor ablations^18–20^. However results from Substrate and Trigger Ablation for Reduction of Atrial Fibrillation Trial (STAR AF II) have shown the PVI only was as efficient as PVI plus CAFÉ or additional line ablations in patients with permanent AF^21^. Initial results on rotor-targeted ablation in persistent AF were promising^20^; however much more extensive studies are needed before adopting this strategy as part of the routine of persistent AF ablation^22^.

Regarding results, an important finding was that the mean total procedure time was shorter in the cryo group than in the RF group (124 vs. 141 minutes, P < 0.001), as was the left atrial dwell time (the length of time the catheter was present in the left atrium during the procedure), (92 vs. 109 minutes, P < 0.001). In addition, the mean total fluoroscopy time was shorter in the radiofrequency group than in the cryoballoon group (17 vs. 22 minutes, P < 0.001). The procedures in FIRE AND ICE were done in high volume centres, with more than 100 cases of AF ablation per year. RF ablation and creating a complete line of ablation around the pulmonary veins is a technically demanding procedure and requires a long learning curve. This may be in contrast to the relatively easier cryo ablation which is less operator-dependent. These points must be considered when extrapolating FIRE and ICE results to the real world practice where, for example, more than 80% of AF ablation cases in the United States are performed by operators with less than 25 cases per year and in centres with less than 50 cases per year^23^.

Another significant result was the lower rate of major complications in both study arms. There were no tracheosophageal fistulae in the RF group and only 10 phrenic nerve injuries in the cryo group, with only one persisting as a clinical problem after 2 years follow up^8^. Even with such low rates, one must acknowledge that AF ablation carries a significantly higher risk of complications relative to other invasive electrophysiology procedures^7^. In a large analysis of 93,801 cases of AF ablation done in United States, in-hospital complications were inversely related to the experience of the operator^23^.

**Figure 2.**
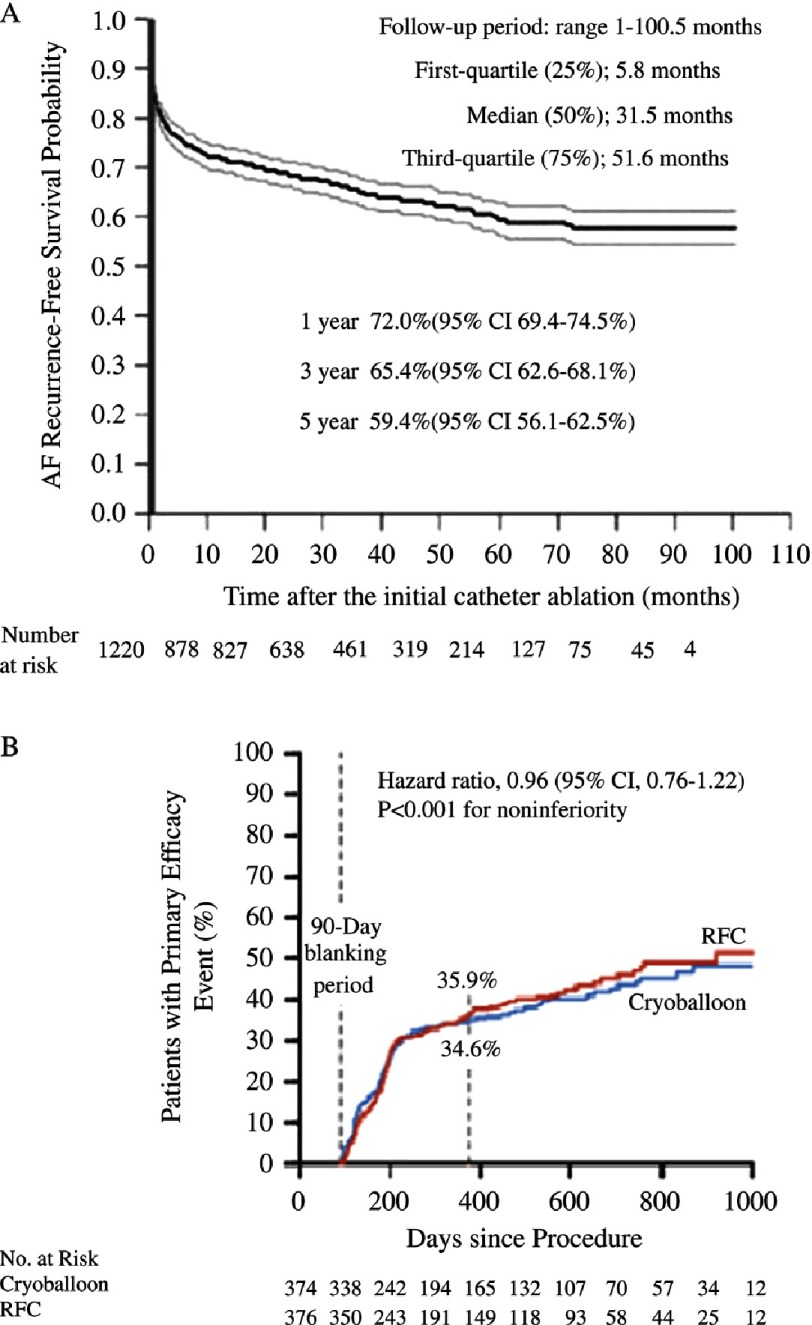
**Panel A** - Atrial fibrillation recurrence–free survival probabilities and 95% confidence intervals (CIs) after the initial catheter ablation (CA); AF recurrence–free survival probabilities 5 years after the initial CA were 59.4%. There is no blanking period. Source: Takigawa et al., Long-Term Effect of CA on PAF. Circ Arrhythm Electrophysiol. 2014;7:267-273. **Panel B** - The 90-day landmark analysis of the primary efficacy end point. The trial confirmed the noninferiority of cryoballoon ablation to radiofrequency (RFC) catheter ablation. The first 90 days after the index ablation was the so-called “blanking period”; events during this period were not counted in the determination of clinical failure for the primary end point.

The most striking point regarding FIRE and ICE results was the high recurrence rate in both arms, which is about 35% (Figure 2). Recurrence remains the Achilles heel in AF ablation. In fact, this 35% recurrence rate may even be underestimated as investigators have only tested for AF by clinical symptoms and 24 hour Holter monitoring^8^. Silent AF episodes missed in between those recordings would further increase the rate of recurrence^24^. Moreover, the study population may be more favourable than AF patients ablated in real life as this group of patients were relatively young, with small left atria, and paroxysmal AF. The recurrence rates would be expected to be even higher in older patients with bigger atria and more persistent AF^25,26^.

This high percentage of recurrence during the follow up in this study and in many other large studies should direct the leaders in the electrophysiology community to question the futility of PVI as an effective management strategy in patients with AF^27–31^. The Atrial Fibrillation Follow-up Investigation of Rhythm Managament (AFFIRM) trial which compared rate control versus rhythm control in patients with AF have showed that rhythm and rate control strategies do not differ in long term morbidity and mortality in AF patients^32^. In addition, no large trials have investigated AF ablation effect on mortality in comparison to antiarrhythmic medications. To answer this fundamental question, we will need to wait 2 more years before the long anticipated CABANA trial results are out. The CABANA (Catheter Ablation Versus Antiarrhythmic Drug Therapy for Atrial Fibrillation) (NCT00911508) is multicenter trial, with planned recruitment of 3,000 patients with paroxysmal, persistent or “chronic” AF in North America, Europe, Australia, and Asia, who will be randomly assigned to either antiarrhythmics or catheter ablation. The primary end point of the CABANA trial is all-cause mortality. Finally, the “favourable” reported improvement in symptoms of patients who have undergone AF ablation was never tested against a sham control trial. The SIMPLICITY 3 trial have emphasized the importance of such studies in evaluating the futility of invasive risky procedures^33^.

## What we have learnt?

AF ablation still carries suboptimal results regardless of type of technology with significant complication rates. This notion is true even when AF ablation is performed in highly experienced centres and with optimal patient selection. Thorough understanding of the electrophysiological and pathological basis that cause and maintain AF remains the missing element in our quest for optimal management of AF.
